# Monthly Summary

**Published:** 1891-05

**Authors:** 


*Editor of the American Journal of Dental Science:*

### Monthly Summary.

BLEEDING AFTER EXTRACTION.—*By Theodore F. Chupein, D. D. S., Philadelphia, Pa.*—Cases of this kind are always attended with a certain degree of solicitude. We had recently advised the extraction of three remaining teeth with the view of inserting a set of artificial substitutes. One of these teeth was the right upper second molar, from which the gum had receded so much that it seemed to be held in place only by the gum-tissue, there being no alveolus. It has been our experience that cases of this kind, when bleeding succeeds extraction, are the most difficult to control, as there is no socket wherein to pack styptics. Dr. Atkinson says that the cause of the bleeding, when it comes from the blood-vessels at the bottom of the socket, is due to the fact that the mouth of the blood-vessels is caught in the socket, and is thus unable to close on themselves. He recommends in such cases that the best thing to do is to take a burr and burr the end of the socket a little larger, so as to allow the sides of the open-mouthed vessels to be free from the wall, when they will contract and the hemorrhage cease.

The case to which we alluded bled from the second upper molar, which had no socket, as we before said, yet the bleeding was so profuse and persistent that the lady was in a fearfully weakened condition from the loss of blood. We had only sent her to have the teeth extracted about 10 o'clock in the morning, but by 8 o'clock at night a messenger came to report the condition of affairs, expressing great alarm at her condition.

We had that morning been reading the paper of Dr. W. L. Roberts "On the internal administration of tannic acid for hemorrhage after tooth extraction," so that we determined to give the remedy a trial. We weighed out *three grains of tannic acid* and gave it to the messenger, with instructions to dissolve it in two-thirds of a tumbler of water, and to give the patient two teaspoonfuls of the solution every five minutes until she had taken three doses, after which to give the same quantity fifteen minutes apart, but if the bleeding ceased after the second or third administration to cease the medicine. The following morning we called to see the patient and found her still very weak and quite pale from the loss of blood. She told us that the bleeding ceased after the second dose of medicine, but that she thought she would die from excessive weakness.

The object of this paper is to call attention to this remedy. Dr. Roberts recommends that *three grains of tannic acid* be dissolved in *one-third of a tumbler of water*, and given in doses such as we have stated, but forgetting at the time whether he had stated one-third or two-thirds of a tumbler of water, we told the messenger to dissolve the drug in two-thirds. In our case the administration proved equally as efficacious in the more homœopathic dose. This is the first and only case in which we have tried the remedy, but its happy termination will induce us to give it more extended trial and note its exhibition. We do not desire, in this article, to "take the wind out of Dr. Roberts' sails," but merely to call attention to his valuable suggestion and bear testimony to its efficacy.—*Dental Office and Laboratory.*

---

The Detection of Abscess.—The diagnosis of an abscess, especially of one near the surface is ordinarily considered so simple a matter that many text books lay down scarcely any rules for the guidance of the surgeon. It is usually simple, yet every now and then we meet with cases in which it is of the utmost importance to know whether pus be present or no, and do what we can we cannot be sure Even an exploratory puncture sometimes fails to give us the desired information, or rather misleads us by giving us reason to believe that pus is not present when it is. In a chapter with this heading, in his excellent "Studies from Old Case Books," Sir James Paget refers to two or three points of considerable importance in the diagnosis of this condition, which are sometimes overlooked or forgotten by the surgeon, but which, if remembered, might save him from an occasional embarrassing blunder. Among these is the condition called by the author, "central softening, such as gives a feeling of softness or easy yielding to pressure in some small portion of a swelling, or of any part of which the rest is firmer or more tense than is natural." This central softening is to be detected by drawing the finger slowly and with even pressure over the suspected part, and when it reaches the point, if there be any, where pus has begun to form a slight yielding will be felt. This soft spot is sometimes of very small area, but its presence is diagnostic and is enough, in the words of the writer, "to justify puncture, incision, scraping, or any other operation that in each case may be deemed best." This sign is especially valuable in suppurating lymphnodes, yet there are here at least two sources of error which are to be avoided in judging of the evidence furnished by the finger. The first of these is in the case of a cluster of inflamed lymph-nodes in which the spaces between the indurated glands may simulate the central softening. The other is in the case of acute cervical lymphadenoma in which the rapid softening of the tumor imitates abscess formation, a condition which, the author confesses, once so far deceived him as to lead him to puncture the swelling in the expectation of letting out pus. This diagnostic point is seldom mentioned in works on surgical diagnosis, and is usually learned only by practice, sometimes, we doubt not, never learned, but it is a most practical one. It is of special utility in cases of phlegmon seated near the surface yet not exactly superficial, which causes perhaps no tumefaction but only a little redness and some tenderness. Sir James Page mentions several cases of this sort which he found recorded in his note-books, in which there was apparently a superficial phlegmon. Careful search with the finger-tip revealed a spot of central softening, not raised or pouting, which, on incision, proved to be the beginning of a fistulous track, several inches in length, passing down to a deeply seated abscess. The author touches on many other interesting points in this chapter, which we are obliged to pass over, but which are well worthy of consideration and of storing in the memory for future need.—*Medical Record.*

---

HOW TO KEEP NEEDLES AND SMALL INSTRUMENTS FROM RUSTING.—Dr. R. H. M. Dawbarn writes to the New York *Medical Journal* regarding the above subject: "For the past year I have been pleased with the results of a new plan—new to me, that is, though very probably not to others. This is simply to keep my needles in alcohol. For extreme safety against rust I use absolute alcohol, but the commercial article would probably be efficient. At least, some needles that I have kept in common alcohol for a month as an experiment are as bright as ever. Upon buying the needles I immerse them in benzine to remove grease. Then, after running them through a towel, I plunge the point (a cutting-edge Hagedorn) into a bit of cork the size of a pea—to avoid dulling from jolting—and finally, with their corks, they are put and kept in a wide-mouthed, glass stoppered bottle filled with absolute alcohol. After use, I sew through a thick, wet, soapy towel repeatedly, cleanse the eye with a thread, immerse in benzine, and finally replace in the alcohol. This last is certainly an efficient disinfectant, besides being an excellent protector against rust. By the bye, I long ago gave up using (save in bowel work) any other than Hagedorn self-threading needles, which are a decided comfort, and, when properly made, do not cut the thread."—*Medical Rccord.*

---

HOW TO LOOSEN GLASS STOPPERS.—The *Pottery & Glassware Rep.* states that some one of the following methods is certain to prove effective:

1. Hold the bottle or decanter firmly in the hand or between the knees, and gently tap the stopper on alternate sides, using for the purpose a small piece of wood and directing the strokes upward.

2. Plunge the neck of the vessel in hot water, taking care that the water is not hot enough to split the glass. If the stopper is still fixed, use the first method.

3. Pass a piece of lint around the neck of the bottle, which must be held fast while two persons draw the lint backwards and forwards.

4. Warm the neck of the vessel before the fire and when it is nearly hot the stopper can be removed.

5. Put a few drops of oil around the stopper where it enters the glass vessel which may then be warmed before the fire. Then apply process No. 1. If the stopper still continues immovable, repeat the above process until it gives away, which it is almost sure to do in the end.

6. Take a steel pin or needle and run it round the top of the stopper in the angle formed by it and the bottle. Then hold the vessel in your left hand and give it a steady twist toward you with the right, and it will very soon be effectual. If this does not succeed, try process No. 5 which will be facilitated by it.—*Med. and Surg. Reporter.*

---

RISKS OF COCAINE INJECTIONS.—The *Medical Press*, November 5, 1890, says that two warning cases are reported from France. In one of them, which occurred at Lille, the patient died, and the dentist who gave the injection was acquitted of neglect, but condemned for practicing medicine without qualification. In the other, which occurred at Paris, the patient was with great difficulty brought round by hypodermic injections of ether. The cocaine injection was also made in this case by a dentist.

In another place the same journal says that the Lille dentist, who was charged with causing the death of a girl by injecting cocaine in order to procure anæsthesia, has been sentenced to a fine of fifteen francs for breach of the law regulating the practice of medicine, the judgment endorsing the view that cocaine is an anæsthetic which requires to be used with prudence, and cannot legally be administered by other than a qualified medical man.—*Med. and Surg. Reporter.*

---

SIMPLE TREATMENT OF INGROWN TOENAIL—The Pittsburg "Medical Review, February, 1891, cites Dr. Puerckhausr (from the "Munchner Med. Wochenschrift) as recommending a simple and at the same time competent treatment for ingrown toenail.

A forty per cent. solution of potassa is applied warm to the portion of the nail to be removed. After a few seconds the uppermost layer of the nail will be so soft that it can be scraped off with a piece of sharp-edged glass; the next layer is then moistened with the same solution, and scraped off; this must be repeated until the remaining portion is as thin as a sheet of paper, when it is seized with a pincette and lifted from the underlying soft parts and severed from the other half. The operation does not require more than half an hour's time, is painless and bloodless, while the patient is delivered from his suffering without being disabled even for an hour.—*American Prac. and News.*

---

IODINE IN THE TREATMENT OF WARTS.—Given to adults, in doses of ten drops twice daily, the tincture of iodine has proved very successful in preventing the further development of warts and causing those already present to disappear. Dr. Imossi remarked, in the ten cases which had come under his observation, that the treatment produced striking emaciation, a fact which he has since turned to account in the treatment of obesity. He has succeeded in procuring a diminution of weight of about twelve pounds in three weeks.—*The London Medical Recorder.*

---

A SAFE LOCAL ANÆSTHETIC.—If we are to believe the statement of Dr. C. T. Meaker, of Carbondale, Pa., in the *Items of Interest*, a few drops of a five per cent. solution of carbolic acid in water, injected under the gum on each side of a tooth to be extracted, makes an excellent and safe local anæsthetic. Its effect, he claims, is almost instantaneous, and is particularly marked when there is swelling and inflammation about the tooth. As only one drop of carbolic acid is contained in twenty drops of the solution, the quantity used is too small to cause any constitutional disturbance.

ELECTRICITY IN EXTRACTION.—The last number of the *Journal of the British Dental Association* contains a letter written by Dr. H. F. Briggs, giving his experience in having a tooth extracted with electricity as an anæsthetic. He says that the sensation during the operation was like being "the central figure" at a miniature execution by electricity, and that he not only felt the pain of the tooth being taken out, but pain about the head, face and arms as well. To this was added the helpless feeling of not being able to let go the handles while the current was on. He informed the dentist that it hurt a good deal; but the dentist replied: "With due deference to you, I must say it didn't." He says that he would much prefer having a tooth extracted without an anæsthetic of any kind than submit again to the electricity.

---

PHOSPHATE FILLINGS.—While valuing phosphate fillings, especially in the preparatory treatment of children's teeth, and as a non-conducting flooring under metallic fillings, I never insert them in deep cavities of living teeth without applying on the region overlying the pulp a protective film of mastic or carbolized resin. Such a proceeding reduces to a minimum the pain often produced by the introduction of the filling, and, I believe, prevents the ill-effects which otherwise may arise from the near contact with the pulp of the cement.—*Dr. L. Matheson, in The Items of Interest.*

---

DARK JOINTS.—Dr. W. W. France says, in the *Items of Interest*, that he packs the joints of his vulcanite plates with about a quarter of a sheet of gold foil, and that he has never had a dark joint since he commenced its use. Where the space is small, one thickness of the gold pressed in with the edge of a penknife blade is quite sufficient. Tin foil will do equally well.

